# The Relationship Between Epilepsy Control and the Duration of Nighttime Sleep and Afternoon Siesta

**DOI:** 10.7759/cureus.39266

**Published:** 2023-05-20

**Authors:** Mohammed Al-Abri, Abdullah Al Asmi, Syed Rizvi, Talal Al-Mashaikhi, Haifa Al Abri, Hiya Al-Majrafi, Arunodaya R Gujjar

**Affiliations:** 1 Clinical Physiology, Sultan Qaboos University, Muscat, OMN; 2 Neurology, Sultan Qaboos University, Muscat, OMN; 3 Family Medicine and Public Health, Sultan Qaboos University, Muscat, OMN; 4 Clinical Physiology, Sultan Qaboos University Hospital, Muscat, OMN

**Keywords:** people with epilepsy, oman, obstructive sleep apnea, sleep measurement, siesta, antiseizure medications, epilepsy, seizure, sleep

## Abstract

Context: The association between epilepsy control and the duration of sleep among people with epilepsy (PWE) is not well studied in Middle Eastern countries such as Oman.

Aims: To describe the sleep habits of PWE in Oman and explore the association of their sleep habits at night and afternoon siesta with the level of seizure control achieved and antiseizure medications (ASMs) consumed.

Methods: The subjects of this cross-sectional study were adult epilepsy patients attending a neurology clinic. Their sleep parameters were measured for one week using actigraphy. Home sleep apnea testing for one night was conducted to rule out obstructive sleep apnea (OSA).

Results: A total of 129 PWE completed the study. Their mean age was 29.8 ± 9.2  years, and their mean body mass index (BMI) was 27.1 kg/m^2^. There was no significant difference between the people with controlled and uncontrolled epilepsy as regards the duration of night sleep or afternoon siesta (p = 0.24 and 0.37, respectively). There was also no significant correlation between their nighttime sleep duration, afternoon siesta, and the number of ASMs they consumed (p = 0.402 and 0.717, respectively).

Conclusion: The study revealed that the sleep habits of PWE with uncontrolled epilepsy who consumed more ASMs were not significantly different from those with controlled epilepsy who consumed fewer ASMs.

## Introduction

The intimate relationship between sleep and epilepsy has long been recognized [1], but the association between seizure control and sleep is not well understood. A healthy sleep-wake rhythm depends upon maintaining a homeostatic balance between the tendency, need, and duration of sleep in accordance with one’s internal circadian rhythm. Together, these act to inhibit the bodily functions associated with wakefulness and determine the timing and duration of restorative sleep [2].

The interaction between sleep and epilepsy is a complex process with variable outcomes. It has been found that sleep deprivation in people with epilepsy (PWE) may provoke seizures during sleep, especially during the non-rapid eye movement (NREM) phase [3]. Decreased sleep efficiency (increased sleep stage shifts, entries to wakefulness, and the number and duration of awakenings) has been noted in epilepsy patients [4]. Different anti-seizure medications (ASMs) may have different effects on sleep structure. Pregabalin, carbamazepine, and gabapentin are reported to increase slow-wave sleep, whereas levetiracetam reduces it [5]. Sleep latency is reported to be reduced by phenobarbital, phenytoin, gabapentin, carbamazepine, and clobazam. Phenytoin and phenobarbital are reported to reduce rapid eye movement (REM) sleep, while gabapentin and lamotrigine increase it. Daytime sleepiness increased in PWE taking higher doses of levetiracetam as well as those taking phenobarbital and valproic acid but remained unchanged in those who took topiramate and zonisamide [5]. Lamotrigine is also reported to cause insomnia [6]. Sleep disorders, mainly obstructive sleep apnea (OSA), are a cause of poor seizure control; OSA evaluation is recommended for patients with drug-resistant epilepsy and in patients whose seizures persist despite being on multiple ASMs [7, 8].

Even though the relationship between seizure activities and circadian rhythm is reasonably understood, the relationship between uncontrolled seizure activities, multiple ASMs, and the sleep habits of PWE remains underexplored. Furthermore, manifestations of a person’s epilepsy may be different in communities with different sleeping cultures, such as in the Arab world, where afternoon siestas are ubiquitous and culturally embedded [8]. Our study, conducted in Oman, aimed to describe the sleep habits of people with epilepsy (PWE), explore the association between these habits, particularly afternoon siesta and sleep duration at night, and study the relationship between these with the level of seizure control and the number of ASMs consumed.

## Materials and methods

Study design and sample

The data collection for this cross-sectional descriptive study was conducted from November 1, 2018, to July 20, 2020. The participants were patients attending the epilepsy clinic at the neurology department of Sultan Qaboos University, a tertiary teaching hospital in Muscat, Oman. All adult patients (> 18 years) of both sexes with a confirmed diagnosis of epilepsy who consented to participate were included. We defined controlled epilepsy as where the patient had 0-one seizure in the preceding 12 months. Uncontrolled epilepsy was defined as having ≥ two seizures in the preceding 12 months.

Patients who had mental instability, were unable or unwilling to wear an actigraphy device to measure sleep patterns, or could not follow home sleep study instructions were excluded from the study. Shift workers, frequent travelers, and pregnant and breastfeeding women were also excluded. The presence and absence of seizure episodes were identified as reported by the patient.

Sleep measurement

An actigraphy watch (SOMNOwatch™ plus, SOMNOwatch®, Germany, 2014) was the actigraphy device selected for this study. The participants were requested to wear the device 24 hours a day for seven days and were provided with a diary to record their daily sleeping hours and wakefulness. They were also asked to run their daily activities as usual but avoid traveling across time zones during the seven-day period. Patients who were unable to wear the device round-the-clock for one full week were excluded from the study. Actigraphy recordings were analyzed, and timings of sleep and wake-up were obtained. The duration of nocturnal sleep and afternoon siestas were also documented. Night and day sleep durations were categorized based on the American National Sleep Foundation recommendation: normal (seven to nine hours), short (< seven hours), and long (> nine hours) [9]. Afternoon siestas were categorized as short napping (≤ one hour) and long napping (> one hour) [10].

Home sleep study

To exclude a potential confounder, viz., obstructive sleep apnea, we provided all consenting subjects with home sleep study equipment, which is the American Academy of Sleep Medicine (AASM) level III (SOMNOtouch™, SOMNOmedics, Germany) for one night, with instructions on its usage. Sleep study output was manually scored by an experienced polysomnographer, duly checked, and reported by a certified sleep physician. The severity of OSA was classified according to the apnea/hypopnea index and scored based on American Academy of Sleep Medicine (AASM) guidelines [11].

Statistics

The database for the study was created in IBM Statistical Package for Social Sciences (SPSS) Statistics for Windows, version 23.0 (IBM Corp., Armonk, N.Y., USA). The participating PWE were categorized into two groups based on the number of seizures they had in the preceding 12 months. Those groups were "controlled epilepsy" and "uncontrolled epilepsy". The participants were also divided into two groups according to the number of different ASMs they were using: 0-1 ASM users and ≥ 2 ASM users. On the basis of the nighttime sleep duration, the participants were again classified into three groups: < seven hours, seven to nine hours, and > nine hours. Finally, the participants were classified based on their afternoon siesta habits into three categories: no siesta, short siesta (< one hour), and long siesta (> one hour).

A chi-square test was used to find the association between the categorized variables. The Kolmogorov-Smirnov test was applied to test the normality of continuous variables. To test the significance of the difference between the mean values of the two groups, a Student’s t-test was used where the distribution pattern was found to be normal; otherwise, the Mann-Whitney U test was applied. A p-value of ≤ 0.05 was taken as significant.

## Results

**Figure 1:**
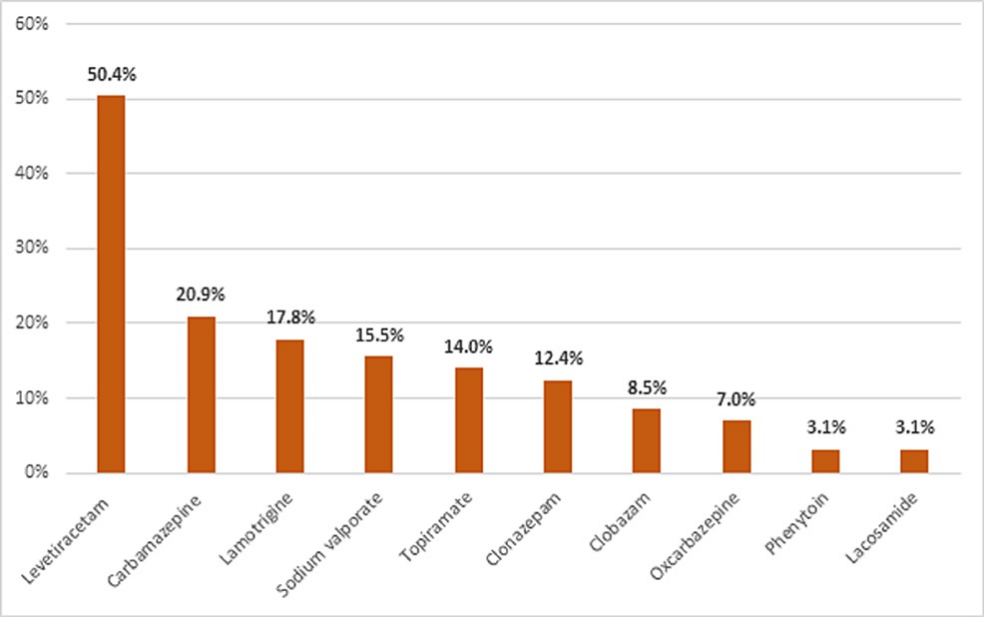
Antiseizure medications consumed by the participants (N = 129) with the mean frequency of use of each.

Out of the 250 PWE who attended the epilepsy clinic regularly and consented to participate in the study, only 129 patients (76 men and 53 women) were included in the study based on the exclusion criteria as described above in the method section. The mean age of the male participants was 30.61 ± 9.79 years, and their mean BMI was 27.15 ± 5.76 kg/m2. The corresponding values for the females were age: 28.53 ± 8.16 years, and BMI: 27.24 ± 7.41 kg/m2. More demographic details are given in Table 1. The details of the ASMs consumed by our participants are provided in Figure 1.

**Table 1:** Comparative demographics and clinical features of male and female patients and those with controlled and uncontrolled seizures. * Mann-Whitney U test applied, due to non-normal behavior of the data. BMI: body mass index; AHI: apnea-hypopnea index

Characteristic	Sex/ Seizure status	n	Mean	SD	p-value
Age (years)	Male	76	30.61	9.79	0.207
	Female	53	28.53	8.16	
	Controlled	57	30.56	9.78	0.38
	Uncontrolled	72	29.11	8.69	
BMI (kg/m^2^)	Male	59	27.15	5.76	0.94
	Female	40	27.24	7.41	
	Controlled	42	27.36	6.51	0.82
	Uncontrolled	57	27.06	6.46	
Sleep duration (hours)	Male	75	7.20	1.41	0.174
	Female	53	7.57	1.65	
	Controlled	57	7.18	1.56	0.242
	Uncontrolled	71	7.49	1.48	
Epilepsy duration (years)	Male	76	10.89	7.71	0.049*
	Female	53	13.04	7.44	
	Controlled	57	12.58	7.87	0.231*
	Uncontrolled	72	11.14	7.46	
AHI	Male	76	12.72	12.81	< 0.001*
	Female	53	5.97	5.97	
	Controlled	57	11.41	13.28	0.451*
	Uncontrolled	72	9.00	8.72	
Afternoon siesta (minutes)	Male	74	56.1	62.1	0.105*
	Female	53	79.2	76.9	
	Controlled	57	57.74	62.39	0.372*
	Uncontrolled	71	71.97	74.13	

Obstructive sleep apnea and epilepsy control

The study revealed significant differences in the indices of apnea-hypopnea (AHI) between the males (12.72) and females (5.97) in the cohort (p < 0.01). AHI was not significantly different between participants with controlled and uncontrolled epilepsy (p = 0.451) (Table 1). There was no significant association between the severity of OSA in patients with controlled or uncontrolled epilepsy (p = 0.191) (Table 2).

**Table 2:** Comparison of sleep parameters between patients with controlled and uncontrolled seizures. OSA: obstructive sleep apnea

Characteristics	Patient category	No. of patients	Seizure status				p - value
			Controlled		Uncontrolled		
			n	%	n	%	
Afternoon siesta	No siesta	51	25	49.0	26	51.0	0.477
	Short siesta	21	07	33.3	14	66.7	
	Long siesta	56	25	44.6	31	55.4	
Patients with obstructive sleep apnea	Normal to mild OSA	108	45	41.7	63	58.3	0.191
	Moderate to severe OSA	21	12	57.1	09	42.9	
Night sleep duration	≤ 6.9 hours	48	25	52.1	23	47.7	0.368
	7.0 – 8.9 hours	57	22	38.6	35	61.4	
	≥ 9.0 hours	24	10	41.7	14	58.3	

Sleep parameters and epilepsy control

The patients with uncontrolled epilepsy tended to sleep longer at night (7.5 ± 1.5 hours) than those with controlled epilepsy (7.2 ± 1.6) (p = 0.242). Those with uncontrolled epilepsy also took longer afternoon siestas, though the difference was not significant (72 ± 74.1 min vs. 57.7 ± 62.4 min; p = 0.372) (Table 1). There was also no significant difference in mean siesta durations between the controlled and uncontrolled PWE (p = 0.477) (Table 2). Nor did cross-tabulation show any significant association between the duration of the siesta and the level of seizure control (Table 2). There was also no significant difference between the controlled and uncontrolled groups in nighttime sleep duration (p = 0.368) (Table 2). Cross-tabulation also did not show a significant relationship between the level of epilepsy control and nighttime sleep durations (Table 2).

Sleep parameters and antiseizure medications

Further, crosstabulation analysis did not show any significant association between the number of ASMs consumed and siesta habits (p = 0.717) or between the number of ASMs and the duration of night sleep (p = 0.402) (Table 3).

**Table 3:** Comparison of sleep parameters of patients who consumed 0–1 or ≥ 2 antiseizure medications (ASM) ASM: antiseizure medication; OSA: obstructive sleep apnea

Characteristics	Patient category		Patients taking ASM				p-value
		No. of patients	0–1		≥ 2		
			n	%	n	%	
Afternoon siesta	No siesta	51	31	60.8	20	39.2	0.717
	Short siesta	21	11	52.4	10	47.6	
	Long siesta	56	30	53.6	26	46.4	
Patients with obstructive sleep apnea	Normal to mild OSA	108	61	56.5	47	43.5	0.955
	Moderate to severe OSA	21	12	57.1	09	42.9	
Night sleep duration (hours)	≤ 6.9 hours	48	25	52.1	23	47.7	0.402
	7.0–8.9 hours	57	36	63.2	21	36.8	
	≥ 9.0 hours	24	12	50.0	12	50.0	

## Discussion

Worldwide, very few studies have evaluated the relationship between sleep duration and epilepsy control. To our knowledge, this is the first study in the Middle East to investigate this phenomenon and the first to study the relationship between epilepsy control and afternoon siestas, an ancient and still-ubiquitous habit in the Arab world.

This study described the sleep habits of a sample of 129 epilepsy patients in Oman and the patterns of their sleep per 24-hour cycle. Our participants slept seven to eight hours at night, similar to the general population of Oman [12]. Nearly two-thirds practiced afternoon siesta, a normal practice in this part of the world [13], the duration of which ranged from less than an hour to more than two hours. We checked for OSA in our patients, a common confounding factor for daytime sleepiness, and found its prevalence to be slightly higher than in the general population of Oman [1, 14]. A meta-analysis of 26 studies from different parts of the world reported a mean OSA prevalence of 33.4% among PWE. We did not find any significant association between AHI and epilepsy control or the number of ASMs taken.

Patients with drug-resistant epilepsy represent about 30% of the total PWE [15]. One study using polysomnography found a 33% prevalence of OSA in this group of patients who were candidates for epilepsy surgery [8]. We did not perform full polysomnography on our participants, as assessing drug-resistant epilepsy was not among our objectives. However, it is a fertile topic for future research among PWE in Oman.

We found no correlation between the duration of nighttime sleep and the number of ASMs consumed by patients. We also found no significant difference in the duration of nighttime sleep between patients who were taking no or a single ASM and those consuming multiple ASMs, though the latter tended to sleep longer at night. Though sleep deprivation and disturbances in sleep are reported to trigger seizures [16], how much sleep deprivation can be considered a risk for uncontrolled epilepsy remains unknown. In our study, people with controlled epilepsy slept marginally less at night compared to those with uncontrolled epilepsy. This might be attributed to the effects of multiple ASMs and frequent seizures among the latter. Most of our patients were taking levetiracetam, known to increase total sleep time and reduce awakening [5].

The strength of our study is that it used an objective method to measure or sample sleep habits and duration. In addition, all participants were screened for OSA using the reliable Level 3 sleep study, though it did not reveal sleep staging or arousal index, which are required details in epilepsy research.

Among the limitations of our study is that it was a single-center experience. A larger sample size and repeating the study in different parts of Oman would have shown a clearer and more generalizable picture of sleep habits among epilepsy patients in the country. The low compliance rate among the participants was an obstacle to achieving the target participation. Variations in sleep habits during the course of just a single week are likely to have impacted the accuracy of the results. However, longer study periods would have severely tested the patience of many participants in wearing the device and writing down their sleep habits for weeks, resulting in more dropouts. The one-week-long sleep sampling did have one benefit, as the same period is adopted by many sleep studies, facilitating interstudy comparisons [17]. Another obstacle was the mismatch between patient-reported and instrument-measured sleep hours, leading us to prefer the instrument to patient recall. Another limitation of our study was that we combined patients with focal and generalized types of epilepsy as a single group; we also did not differentiate between patients with focal epilepsy who had undergone epilepsy surgery and those who did not. Further, our study did not control for the fact that our subjects had been prescribed different ASM doses, combinations, and types, which may have had complex effects on their sleep parameters and seizure proneness. Furthermore, we did not control for the effect of seizure events that occurred during the study period (one week per patient) and how such events might affect sleep architecture.

## Conclusions

This study found that epilepsy patients in Oman with uncontrolled epilepsy and those who were taking multiple antiseizure medications had sleep habits (including nighttime sleep and afternoon siestas) similar to those of the general population. It was also found that the use of multiple antiseizure medications was associated with a longer duration of sleep at night.
